# Multi-omics profiling in spinal muscular atrophy (SMA): investigating lipid and metabolic alterations through longitudinal CSF analysis of Nusinersen-treated patients

**DOI:** 10.1007/s00415-025-12909-4

**Published:** 2025-02-04

**Authors:** Martina Zandl-Lang, Thomas Züllig, Michael Holzer, Thomas O. Eichmann, Barbara Darnhofer, Annette Schwerin-Nagel, Joachim Zobel, Harald Haidl, Ariane Biebl, Harald Köfeler, Barbara Plecko

**Affiliations:** 1 Research Unit of Analytical Mass Spectrometry, Cell Biology and Biochemistry of Inborn Errors of Metabolism, Department of Paediatrics and Adolescent Medicine, Medical University of Graz, 8036 Graz, Austria; 2 Institute of Molecular Biosciences, University of Graz, 8010 Graz, Austria; 3 Division of Pharmacology, Otto Loewi Research Center for Vascular Biology, Immunology and Inflammation, Medical University of Graz, 8010 Graz, Austria; 4 Core Facility Mass Spectrometry, ZMF, Medical University of Graz, 8036 Graz, Austria; 5 Division of General Pediatrics, Department of Pediatrics, Medical University of Graz, 8036 Graz, Austria; 6 Department of Paediatrics, University Children’s Hospital Linz, 4020 Linz, Austria

**Keywords:** Mass spectrometry, Biomarker, Omics, Lipid metabolism, Neuromuscular

## Abstract

Spinal muscular atrophy (SMA) is a rare neuromuscular disease caused by biallelic mutations in the SMN1 gene, leading to progressive muscle weakness due to degeneration of the anterior horn cells. Since 2017, SMA patients can be treated with the anti-sense oligonucleotide Nusinersen, which promotes alternative splicing of the SMN2 gene, by regular intrathecal injections. In this prospective study, we applied metabolomic, lipidomic, and proteomic analysis to examine sequential CSF samples from 13 SMA patients and controls. This multi-omic approach identified over 800 proteins and 400 small molecules including lipids. Multivariate analysis of multi-omic data successfully discriminated between the CSF derived from SMA patients and control subjects. Lipidomic analysis revealed increased levels of cholesteryl esters and lyso-phospholipids, along with reduced levels of cholesterol and phospholipids in the CSF of SMA patients as compared to healthy controls. These data, combined with results from functional assays, led us to conclude that SMA patients exhibit altered levels and function of high-density-lipoprotein (HDL)-like particles in the CSF. Notably, Nusinersen therapy was observed to reverse disease-specific profile changes toward a physiological state, potentially explicable by restoring HDL function.

**Supplementary Information:**

The online version supplementary material available at 10.1007/s00415-025-12909-4.

## Introduction

Spinal muscular atrophy (SMA) is a rare autosomal recessive neuromuscular disease caused by biallelic mutations in the survival motor neuron (*SMN1*) gene. All SMA patients lack a functional *SMN1* gene, but retain one or more copies of the highly homologous *SMN2* gene, harboring a single-nucleotide change. This single-nucleotide exchange results in the exclusion of exon 7 from approximately 90% of the mature SMN2 transcripts, leading to an unstable and rapidly degrading protein [1]. The SMN1 and 2 protein are ubiquitously expressed in the cytoplasm and highly expressed in the spinal cord, where they are essential for motor neuron survival. SMA has been known as the most common genetic cause of infant mortality [2]. Untreated SMA patients display a wide range of clinical manifestations including progressive muscle weakness due to the loss of alpha motor neurons in the brain stem and spinal cord. The disease severity inversely correlates with expression levels and copy numbers of the SMN2 protein. All SMA types are progressive and depending on the age of onset and motor function achieved, SMA has been subdivided into five clinical subtypes [2]. More recent clinical SMA classifications are based on functional abilities into non-sitters, sitters, and walkers. The distinction between non-sitters and sitters is defined by the ability to sit unsupported for 3–10 s, whereas walkers are able to walk independently for at least five steps without arm support [3, 4].

SMN is highly expressed during embryogenesis, probably particularly necessary for neuronal cell differentiation, and its expression rapidly decreases over lifetime [5]. SMN plays an overarching role in gene transcription and protein translation, mitochondrial function, and cytoskeletal dynamics [6]. Specifically, SMN is involved in several RNA metabolic processes, including the synthesis of uridine-rich spliceosomes, assembly of spliceosomal small nuclear ribonucleoproteins (snRNP), and pre-mRNA splicing. In addition, the involvement of SMN in immunoregulatory processes and oxidative stress response, especially in macrophages, has been discussed [7]. In case of reduced SMN protein, microglia exhibit an increased inflammatory profile that negatively affects motor neuron survival. The role of the SMN protein extends beyond the maintenance of motor neurons as in SMN ko iPSC-derived microglia an altered morphology with increased migration and phagocytic activity has been observed [8].

To date, three disease modifying therapies (DMT) have been licensed, leading to increased SMN protein levels by different mechanisms and radically changing the natural course of the disease [6]. In 2016, the FDA approved the first drug, Nusinersen, as an anti-sense oligonucleotide (ASO) treatment of SMA, which increases SMN expression through alternative splicing of SMN2 pre-mRNA. Nusinersen is administered intrathecally and leads to alternative splicing of the SMN2 gene and significant improvement of motor function has been achieved, if treatment is applied early. In 2019, onasemnogene abeparvovec, a gene therapy applied by a one-time intravenous infusion of an adeno-associated virus vector for SMN1 correction has been approved by the FDA for patients with SMA below 2 years of age with biallelic mutations of the SMN1 gene and up to three copies of the SMN2 gene [9]. Finally, in August 2020, risdiplam has been approved by the FDA as an oral small molecule splicing modifier for all types of SMA [10].

Response to either of these DMT is assessed by motor scores such as the Children’s Hospital of Philadelphia Infant Test of Neuromuscular Disorders (CHOP INTEND) or the Revised Upper Limb Module (RULMS), both relatively time-consuming and highly dependent on patient cooperation [11, 12]. There is an obvious lack of a valid biomarkers for SMA that may monitor or even predict therapeutic response across different SMA disease types and age groups. During treatment with Nusinersen, CSF is routinely withdrawn before each intrathecal administration, providing an opportunity to investigate these samples for metabolic and biochemical aberrations through multi-omics profiling.

Multi-omics approaches, performed by mass spectrometric (MS) analysis, offer the potential to provide an accurate and comprehensive readout in the analysis of rare diseases. It has been shown that combining multiple omics techniques with bioinformatic analyses may increase the diagnostic yield, especially in rare diseases, and result in improved and adapted patient care [13]. Previous studies on an SMA mouse model as well as clinical -omics studies on SMA patients revealed alterations in the proteome and metabolome of plasma and CSF as compared to controls [1, 6, 14, 15]. Additionally, SMA has been associated with dyslipidaemia including changes in the fatty acid metabolism [16, 17]. Specifically, it was shown that children with severe forms of SMA (type I), but not older patients with milder forms of SMA (type II and type III) exhibit significant abnormalities in plasma fatty acid levels [18].

The aim of our study was to further investigate alterations of lipid and amino acid metabolism in CSF of SMA patients as well as the individual impact of treatment with Nusinersen on their CSF metabolite and protein profiles. Following ethics approval, we performed omics-analyses in CSF samples of 13 SMA patients, collected along routine procedures as well as from control patients. Through the application of three MS-based omics techniques (proteomics, metabolomics, and lipidomics), we were able to detect metabolic changes before and during Nusinersen therapy compared to age- and sex-matched controls, which help to shed light on potential, disease-associated pathomechanisms. To the best of our knowledge, this study is the first applying extensive lipidomics, metabolomics, and proteomics analysis on an SMA patient cohort.

## Patients and methods

Samples of SMA patients and controls were collected following informed consent at two tertiary-care centers along routine procedures. CSF samples of SMA patients were taken from volumes that had to be withdrawn before administration of Nusinersen; plasma samples were taken along placement of the venous access for sedation. SMA types were classified according to age of onset.

### Chemicals

All chemicals used for sample preparation (protein, metabolite, and lipid extraction) were purchases from Merck (Darmstadt, Germany). Pierce BCA Protein Assay Kit was purchased from Thermo Fisher Scientific, Waltham, USA.

### CSF sample collection and sample characteristics

CSF samples were taken from 13 symptomatic patients (7 male, 6 female) with SMA type I (*n* = 5), type II (*n* = 7), and type III (*n* = 1) along intrathecal treatment with Nusinersen, compared to 14 age- and sex-matched control individuals (ctrl). Patients and/or their legal guardians were asked for informed consent prior to the spinal tap performed according to the study protocol. Ctrl samples were taken after informed consent in the context of routine diagnostic procedures which included 0.5–1.0 mL of additional CSF. CSF samples were stored at -80 °C prior analysis.

### Metabolomics and lipidomics: sample extraction

For metabolomics analysis, a 4:1 volume of ice-cold methanol was added to 100 µL of CSF and vortexed for 15 s as described previously [19]. The sample was then allowed to precipitate at 4 °C for 60 min, followed by centrifugation at 12,000 rpm, for 10 min, at 4 °C. The resultant supernatant was transferred into a clean Eppendorf tube and concentrated under a stream of dry nitrogen gas at room temperature. The resultant plasma and CSF sample pellet were resuspended in ACN/water 8:2 (v/v) to the original volume and either subjected to MS analysis or immediately placed at − 80 °C until further analysis. For lipidomics analysis, the liquid extraction protocol with methyl-tert-butyl ether (MTBE) was applied [19]. In short, 1.5 mL methanol and 5 mL MTBE were added to 100 µL of CSF in glass test tubes; 10 µL of 0.5 mM PE 24:0 was added as extraction control. After incubation and addition of 1.25 mL deionized water, the mixture was centrifuged for 5 min at 2000×*g* and the upper phase was transferred to a new test tube. The lower aqueous phase was re-extracted with 2 mL of the upper phase of MTBE/methanol/deionized water 10:3:2.5 (v/v/v). The upper phases were combined, evaporated in a vacuum centrifuge, and dissolved in 500 µL chloroform/methanol 1:1 (v/v) for storage at − 80 °C. For MS analysis of the lipidome, samples were evaporated under a stream of nitrogen and the resultant pellet was resuspended in the original volume, of isopropanol/methanol/water (95:5:5), respectively. Normalization across all samples was realized by a BCA protein assay.

### Metabolomics and lipidomics: LC–MS/MS analysis

The MS analysis of each sample’s metabolite and lipid components was achieved as described previously [19]. In short, lipidomics analysis was performed as described previously using Dionex Ultimate 3000 XRS UHPLC (ultra-high performance liquid chromatography)-Orbitrap Velos Pro hybrid mass spectrometer (Thermo Fisher Scientific) operated in data-dependent acquisition mode using an HESI II ion source [19]. Full-scan profile spectra were acquired in the Orbitrap mass analyzer at a resolution setting of 70,000 at m/z 200. For lipidomics MS/MS experiments, the ten most abundant ions of the full-scan spectrum were sequentially fragmented in the ion trap using He as collision gas and centroided product spectra were collected. For metabolomics analysis, a QExactive Focus (Thermo Fisher Scientific) was used measuring in positive electrospray ionization mode, and full-scan spectra were fragmented in a higher energy collisional dissociation (HCD) cell using N_2_ as collision gas. LC–MS/MS parameters applied for metabolomics analysis are listed in Table 1. This high-resolution technique provided a comprehensive analysis (lipidomics analysis was measured in positive and negative electrospray ionization mode) of the sample’s metabolomics and lipidomics profile. Respective pooled CSF samples were used as quality controls (QC). These QC were repeatedly measured at every 10th position in the acquisition queue to examine and, if necessary, to correct for systematic errors.

**Table 1:** LC–MS/MS parameters used for the metabolomics analysis

Metabolomics	
Column	Acuity UPLC BEH Amide, 2.1 mm × 150 mm, 1.7 µm (Waters Corporation, Milford, MA, USA)
Mobile Phase A	97% ACN + 3% H_2_O + 0.1 mM NH_4_COOH + 0.16% HCOOH
Mobile Phase B	H_2_O + 0.1 mM NH_4_COOH + 0.16% HCOOH
Gradient	Gradient elution started at 1% mobile phase B and increased up to 50% over 14 min. Mobile phase B was reset to start conditions over two minutes and re-equilibrated for 9 min
Injection volume	4 µL
Flow rate	400 µL min^−1^
Ion source parameters	
Spray voltage	3.8 kV
Capillary temperature	300 °C
Sheath gas	35
AUX gas	10
Sweep gas	0
Acquisition of full-scan spectra	m/z 60–900

### CSF cholesterol efflux capacity (CEC) assay

CSF CEC assay was conducted as described previously [20]. In brief, 1 SH-SY5Y human neuroblastoma cells were seeded in 96 well plates using Dulbecco’s modified eagle medium supplemented with 4 mM l-glutamine, 10% fetal calf serum, and 1% penicillin/streptomycin. The cells were immediately labeled with [3H]-cholesterol [2 µCi mL^–1^] for 24 h. On day 2, ABCA1 expression was upregulated in the cells using the liver-x-receptor agonist TO901317 [2 µM] under serum-free conditions for 18 h. Next, cells were exposed to 44% of individual CSF in serum-free conditions for 2.5 h. After exposure, the cell supernatant was collected, floating cells removed by centrifugation, and radioactivity assessed by a scintillation counter upon removal of floating cells. CEC activity was calculated as the percent of [3H]-cholesterol counts in the medium relative to the total counts in medium and cells.

### Proteomics analysis

For proteomics analysis, 5 μg of CSF protein were reduced and alkylated 40 min at 60 °C with final 10 mM TCEP and 40 mM CAA. Samples were digested overnight with trypsin (Promega, enzyme/protein 1:50). Peptides were desalted using SBD-RPS tips [21]. 200 ng per sample (re-dissolved in 2% acetonitrile/0.1% formic acid in water) were subjected to LC–MS/MS analysis. Protein digests were separated by nano-HPLC (Dionex Ultimate 3000, Thermo Fisher Scientific equipped with a C18, 5 µm, 100 Å, 100 µm × 2 cm trap column) and an Acclaim PepMap RSLC analytical nanocolumn (C18, 2 µm, 100 Å, 500 × 0.075 mm) (all Thermo Fisher Scientific, Vienna, Austria). Peptides were focused on the trap column for 5 min at a flow rate of 15 µL min^–1^ with 0.1% formic acid as isocratic solvent. Separation was carried out on the nanocolumn at a flow rate of 300 µL min^–1^ at 60 °C using the following gradient, where solvent A is 0.1% formic acid in water and solvent B is acetonitrile containing 0.1% formic acid: 0–5 min: 2% B; 5–123 min: 2–35% B; 123–124 min: 35–95% B, 124–134 min: 95% B; 134–135 min: 2% B; 135–150 min: 2% B. The maXis II ETD mass spectrometer (Bruker Daltonics, Germany) was operated with the captive source in positive mode with the following settings: mass range: 200–2000 m/z, 2 Hz, capillary 16.000 V, dry gas flow 3 L min^–1^ with 150 °C, nanoBooster 0.2 bar, precursor acquisition control top 20 (collision induced dissociation (CID).

The LC–MS/MS data were analyzed by MaxQuant by searching the public SwissProt human database (11,393,515 residues, 20,467 sequences) and common contaminants [22, 23]. Carbamidomethylation on cysteine and oxidation on methionine were set as a fixed and as a variable modification, respectively. Detailed search criteria were used as follows: trypsin, max. missed cleavage sites: 2; search mode: MS/MS ion search with decoy database search included; precursor mass tolerance ± 10 ppm; product mass tolerance ± 25 ppm; acceptance parameters for identification: 1% PSM FDR; 1% protein FDR. Additionally, a label-free quantification (LFQ) was performed using MaxQuant requiring a minimum of two ratio counts of quantified razor and unique peptides.

Data processing was performed with Perseus software version 1.6.6.0. Data were filtered for decoy hits, contaminants and proteins only identified by modified peptides. After log2 transformation, proteins were filtered for containing at least 80% valid values per group. Missing values were imputed with random values based on the Gaussian distribution. Among a total of 1075 potentially detectable proteins, a large majority (997 proteins) was identified in each group.

### Data analysis and statistics

Data obtained from lipidomics analysis by UHPL–MS/MS analysis underwent lipid identification with the Lipid Data Analyzer (LDA version 2.8.3_2) software followed by the data processing step as described previously [19].

Detected features obtained from metabolomics analysis were (1) compared to our in-house generated target list with m/z and RT matched reference standards (confidence level = 1), or (2) underwent metabolite identification using the Compound discoverer 3.1 (CD) and LDA as described previously [24]. These metabolites were then further compared with the Human Metabolome Database (HMD) (confidence level = 2). Unknown features are marked with confidence level 3. Drug metabolites detected in the metabolome were removed prior to statistical evaluation. In total, 123 metabolites, based on selected confidence levels, have been investigated further. Detected metabolites/features were searched within the Human Metabolome Database. Non-detectable compounds are described as unknown features. For clarification, we categorized the top 25 metabolites identified through MVA into (sub-)categories as listed in HMDB. In univariate analysis (UVA), outliers were removed. A list of all detected compounds including associated confidence levels is enclosed in the Supplement Table 2.

R/Rstudio (4.4/2024.04.2 + 764) was used for data processing. Besides basic functions, the *tidyverse* package was used for data transformation and figure generation [25]. To calculate multivariate statistics, the *lipidr* package was used [26], and for calculating univariate Wilcoxon rank sum test with false-discovery rate (FDR) for multiple comparison correction *rstatix* package was used (https://rpkgs.datanovia.com/rstatix/). The significance levels were indicated with asterisks (*p < 0.05, **p < 0.01, ***p < 0.05). The exclusion of outliers is based on values above the third quartile in addition to three times the interquartile range (IQR) or below the first quantile in addition of three times the IQR (Q3 + 3xIQR/Q1-3xIQR).

## Results

**Fig. 1:**
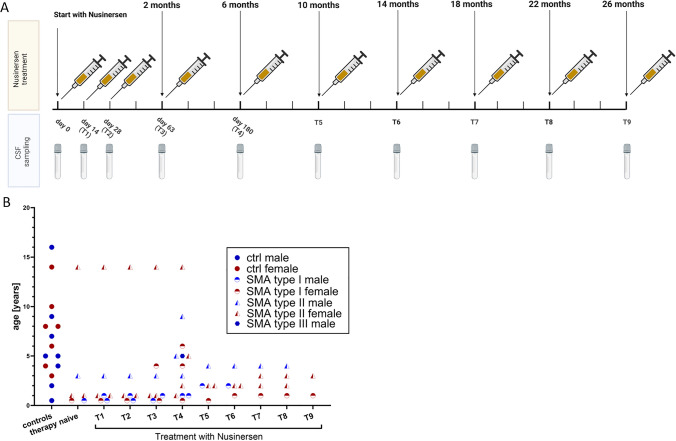
Study design. **A** Intrathecal administration of Nusinersen, dose followed standard treatment protocol (FDA). CSF sampling occurred in parallel with Nusinersen treatment. In total, CSF samples were taken from 13 SMA patients (type I: *n* = 5, type II: *n* = 7, type 3: *n* = 1), including six therapy naïve samples (two SMA type I and four SMA type II) and from 15 age- and sex-matched controls (seven female, six male). Samples on treatment with Nusinersen (T1–T9) were aggregated at timepoint T4

During this study, six therapy naïve (TN) SMA patients (4 female, 2 male) including two SMA type I and four SMA type II patients were initiated on intrathecal treatment with Nusinersen and CSF samples were collected consecutively (T0-T9) (Fig. 1, Supplement Table 1). Seven patients have already been on treatment with Nusinersen at the time of inclusion into this study. Nusinersen therapy followed a standard treatment protocol according to EMA (EMEA/H/C/004312). Timepoint (T4) was selected as our “under therapy with Nusinersen” timepoint, because metabolic and lipid alterations were already detectable, and T4 provided the largest sample cohort of 13 SMA patients, which is crucial for statistical analysis. The SMA cohort at T4 included seven females and six males under therapy including five patients with SMA type I (SMN copy number of two patients = 3 and three patients = 2), seven patients with SMA type II (SMN copy number of five patients = 3 and two patients = 4), and one patient with SMA type III (SMN copy number = 6) (Fig. 1). In addition, CSF samples were collected from 15 sex- and age-matched controls (seven females, eight males), who underwent CSF analysis for exclusion of inflammatory CNS disease.

### CSF of SMA patients indicate a dysregulation of lipid metabolism

**Fig. 2:**
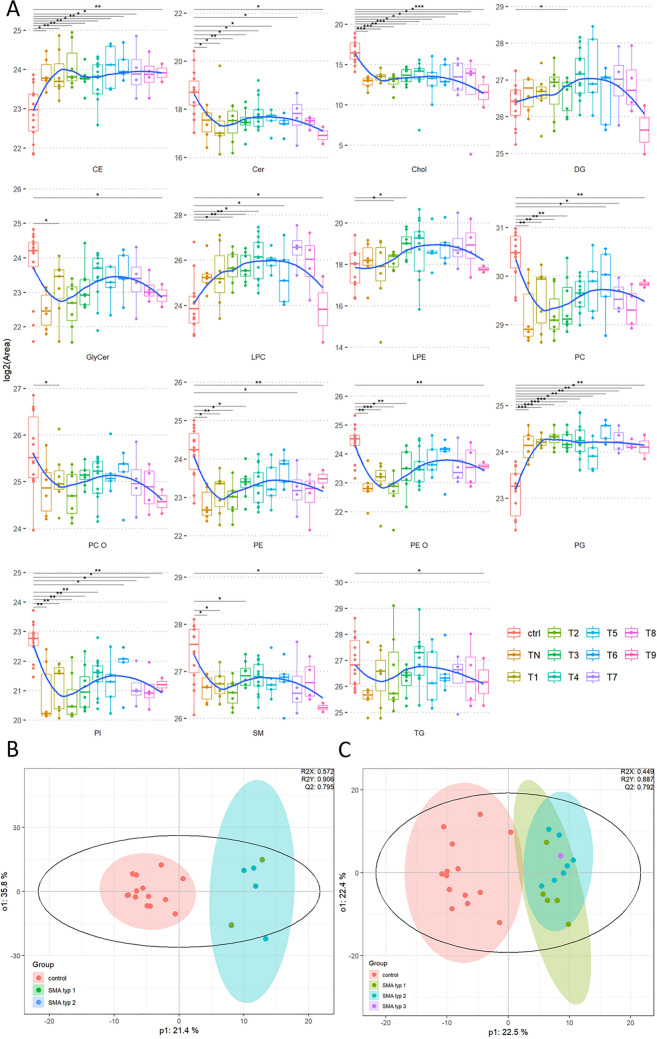
Lipidomics analysis of CSF from SMA patients and controls (ctrl). **A** Wilcoxon sum rank test of lipid classes obtained from the CSF of therapy naïve (TN) SMA patients as well as under therapy with Nusinersen (T1–T9). **B, C** OPLS-DA analysis of lipidomics data obtained from CSF of ctrl and SMA patients (green dots: SMA type I, blue dots: SMA type II, purple dot: SMA type III). **B** Comparison between ctrl and TN SMA patients. **C** Comparison between ctrl and SMA patients under therapy (T4)

Along the characterization of the CSF lipidome of SMA patients, we identified a total of 15 lipid classes using our in-house UHPLC–MS/MS method (Fig. 2A). Analysis revealed global effects between ctrl and SMA patients. All sphingolipid classes detected, including ceramides (Cer), glycosylceramides (GlyCer), and sphingomyelin (SM) were found to be decreased by at least twofold in the CSF of SMA patients. Similarly, triacylglycerols (TG) and the major classes of phospholipids (PL), such as phosphatidylcholine (PC), phosphatidylethanolamine (PE) and phosphatidylinositol (PI), and ether-linked PL of PC (PC-O) and PE (PE-O), were also reduced by about twofold in SMA patients irrespective of treatment. In contrast, phosphatidylglycerols (PG) and lyso-PL, such as LPC and LPE, were increased by ~ 1.9-fold in SMA patients. In addition, the cholesteryl esters (CE) of sterol lipids (ST) were increased in SMA patients by ~ 1.9-fold (irrespective of treatment status) compared to the control group, while free cholesterol (Chol) was decreased by 2.5-fold in TN patients and by 2.3-fold in T4 patients.

**Fig. 3:**
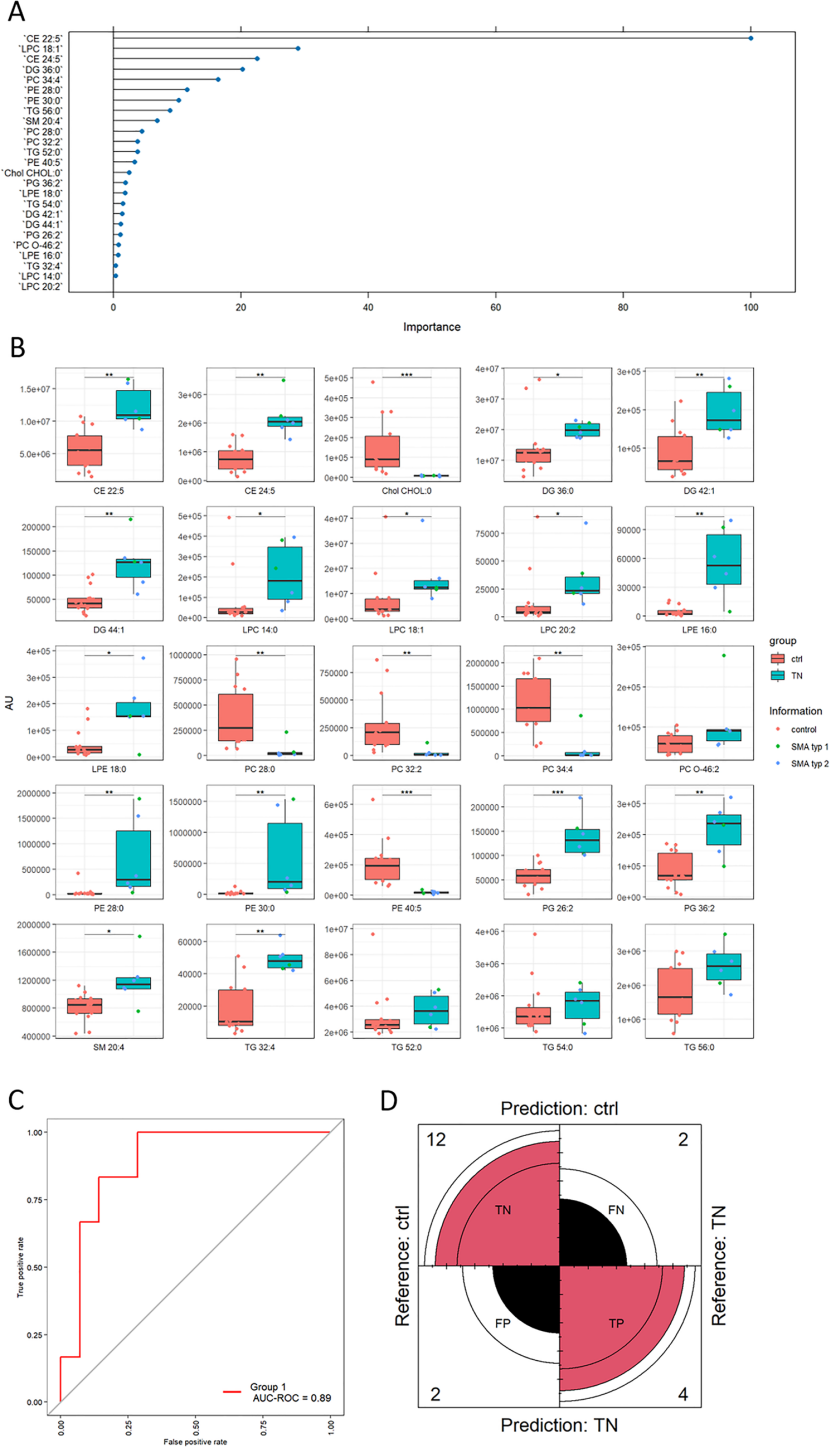
**A** Top 25 lipid species in therapy naïve SMA patients (TN) vs. controls (ctrl) according to molrank in OPLS-DA. A molrank refers to the ranking of molecular features based on their importance in the OPLS-DA and helps to identify which molecular feature contributes most significantly to the separation between different groups in the dataset. Complete list of significantly altered lipid species in TN SMA patients compared to ctrl is provided in a separate table in the supplement section. **B** Box plots from the 25 top lipids derived from OPLS-DA. Red bar: ctrl group, turquoise bar: SMA group with red dots indicating SMA type I and blue dots indicating SMA type II patients **C** ROC plot with an Area under curve (AUC) value of 0.89 indicating a good performance of our model. **D** Confusion matrix generated to evaluate the performance of the MVA model. Out of 20 cases, the algorithm identified 12 true negatives (TN), 4 true positives (TP), two false positives (FP), and two false negatives (FN)

**Fig. 4:**
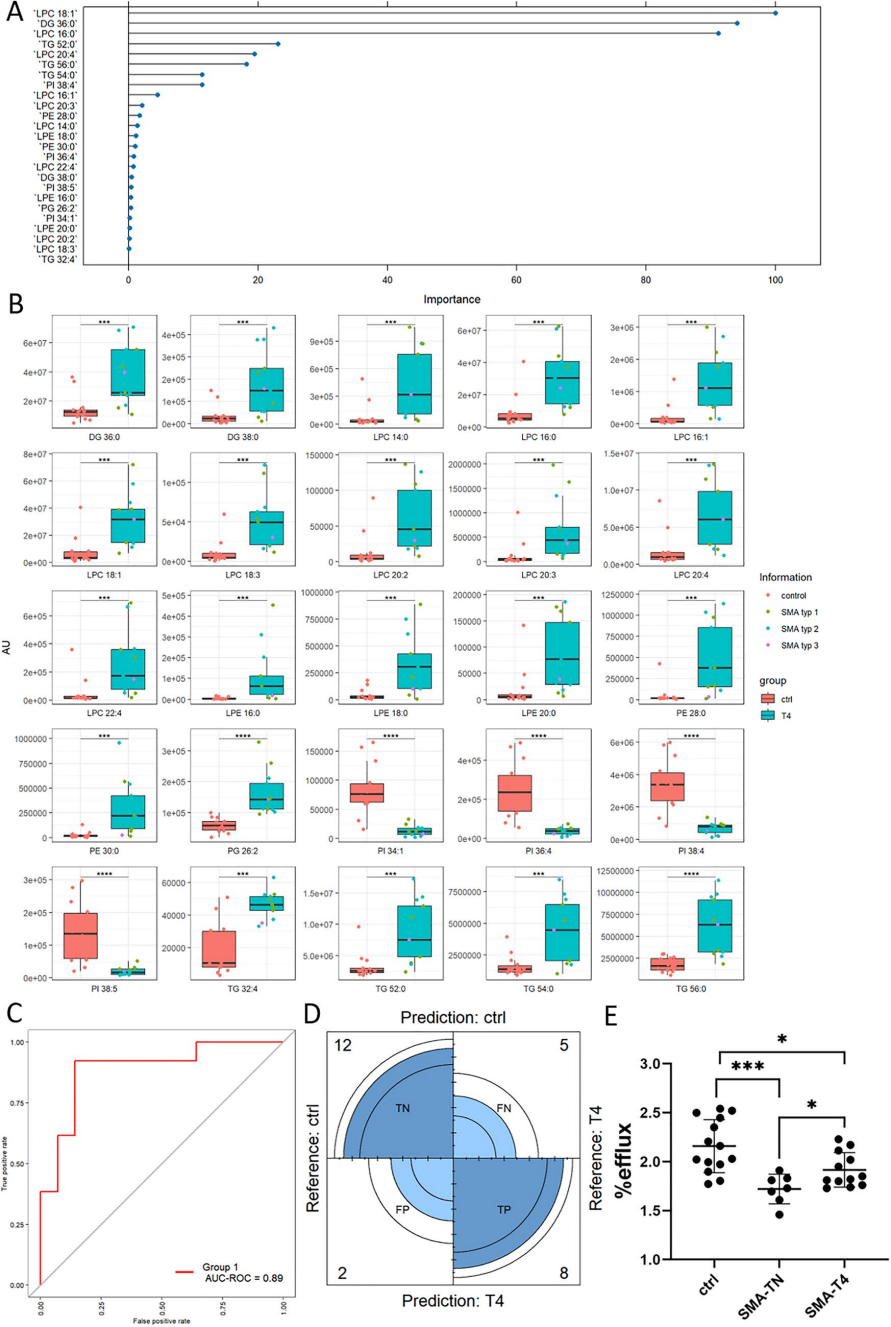
**A** Top 25 lipid species important for discrimination in OPLS-DA of CSF from T4 SMA patients versus ctrl. **B** Univariate analysis using box plots of selected 25 lipid species from discriminant analysis. Red bars indicate ctrl subjects and turquoise bars indicate CSF from SMA patients under therapy (T4) including green dots for SMA type I, blue dots for SMA type II, and purple dots for SMA type III patients, respectively. **C** ROC plot with an area under curve (AUC) value of 0.99 indicating very good performance of our model. **D** Confusion matrix: Out of 27 cases, 12 were identified truly negative, eight true positives, two false negatives, and two false positives. **E** CSF cholesterol efflux capacity (CEC) assay. Following Shapiro–Wilk test for normality testing, data were compared using Welch’s *t* test. CEC was measured at 2.16 ± 0.07 (SEM)% at baseline, 1.72 ± 0.06 (SEM) % in TN SMA patients, and 1.92 ± 0.05 (SEM) % in T4 SMA patients. *p ≤ 0.05; ***p ≤ 0.001

We used an OPLS-DA model to identify relevant differences between lipid species of TN SMA patients and ctrl (Fig. 2B), T4 SMA patients and ctrl (Fig. 2C), and TN SMA and T4 SMA (model not shown) in more detail. We detected a clear separation of TN SMA CSF samples compared to ctrl (Fig. 2B). This separation was diminished by therapy with Nusinersen (T4, Fig. 2C) and was further confirmed by identifying the top 25 lipid species according to molrank in OPLS-DA (Figs. 3, 4). In the CSF of TN SMA patients, we identified 25 lipid species across 11 lipid classes. It was shown that CE and Chol also hold strong discriminative potential. The top hit was CE 22:5, a cholesteryl ester containing docosapentaenoic acid (22 carbon atoms, 5 double bonds). Multivariate analysis (MVA) showed that CE 22:5, as well as CE 24:5 were significantly increased by ~ 1.9-fold in the CSF of TN SMA patients, while Chol was reduced by ~ 2,5-fold. (Fig. 3B). Furthermore, various alterations in PL, such as PCs, PEs, PGs, and their ether-bound- and lysoforms, could be observed. In detail, the lyso-PL (such as LPC 14:0, LPC 18:1, LPC 20:2 as well as LPE 16:0 and LPE 18:0) were significantly elevated in the patient group, whereas the PL PC 28:0, PC 32:2 and PC 34:4 were significantly decreased. On the other hand, PL PE 28:0, PE 30:0, PG26:2, and PG 36:2 were significantly increased in these patients. In addition, SM 20:4, and three TG species (TG 32:4, TG 52:0, TG 56:0) were increased in the CSF of SMA patients compared to ctrl. We observed no discrimination among different SMA types. An ROC plot was generated to evaluate performance of the model applied, showing a good performance with an AUC-ROC value of 0.89 (Fig. 3C). Based on the identified lipid species, a confusion matrix was created to assess the algorithm’s performance. The model correctly identified most cases as true negatives and true positives, with only two cases each identified as false negative and false positive.

When comparing the top 25 lipid species identified in the T4 SMA group versus ctrl compared to those in the TN SMA group versus ctrl, these lipid species originated from fewer lipid classes with LPC as the most prominent lipid class including LPC 18:1 as the top hit. In addition to LPL, such as LPE and LPE, important discriminators included the PL PI, PE and PG, as well as various TG and DG species (Fig. 4A). Similarly, as observed in the TN group lyso-PL (such as LPC 16:0, LPC 16:1, LPC 18:1, LPC 18:3, LPC 20:2, LPC 20:3, LPC 20:4, LPC 22:4 as well as LPE 16:0, LPE 18:0, and LPE 20:0) were significantly increased in the patient group, while PL (such as PI 34:1, PI 36:4, PI 38:4 and PI 38:5) was reduced. The PE species PE 28:0 and PE 30:4 as well as PG 26:2 were also increased in the TN SMA group compared to ctrl, as observed in the TN group. Additionally, DG and TG species were increased in the CSF of SMA patients under therapy compared to ctrl. Similar to the TN patient, there was no indication of different lipid profiles among the three clinical subtypes. Similar to the TN patient group ROC plot showed a good performance for the T4 SMA patients compared to ctrl, with an AUC-ROC value of 0.89. The confusion matrix indicated a solid performance with two false positive and two false negative. Most cases were correctly identified as true negatives and true positives.

Irrespective of treatment status, lipidomics analyses clearly indicated a changed lipid profile in the CSF of SMA patient, with altered ratios of lyso-PL to PL and Chol to CE. This led us to speculate about changes in HDL composition and metabolism in the CSF of SMA patients. To investigate this further, we performed a CSF Chol efflux capacity (CEC) assay to examine HDL function ex vivo (Fig. 4E). Due to low concentrations of HDL-like particles in the CSF and low obtainable CSF volumes in humans, HDL levels cannot be analyzed using standard procedures, such as NMR. Therefore, an indirect functional assay—the CSF CEC assay—reflecting Chol trafficking- was conducted using SH-SY5Y human neuroblastoma cells. At baseline, the CSF CEC in control patients was 2.16 ± 0.07 (SEM)%. In TN SMA patients, CSF CEC was reduced by 20.25% to 1.72 ± 0.06 (SEM) % (*p* ≤  0.001). Treatment with Nusinersen increased CEC in SMA patients by 9.0% to 1.92 ± 0.05 (SEM) % (*p* ≤ 0.05) indicating a move toward physiological conditions.

### Metabolomic profiling reveals distinct CSF metabolome in SMA patients and therapeutic impact of Nusinersen

**Fig. 5:**
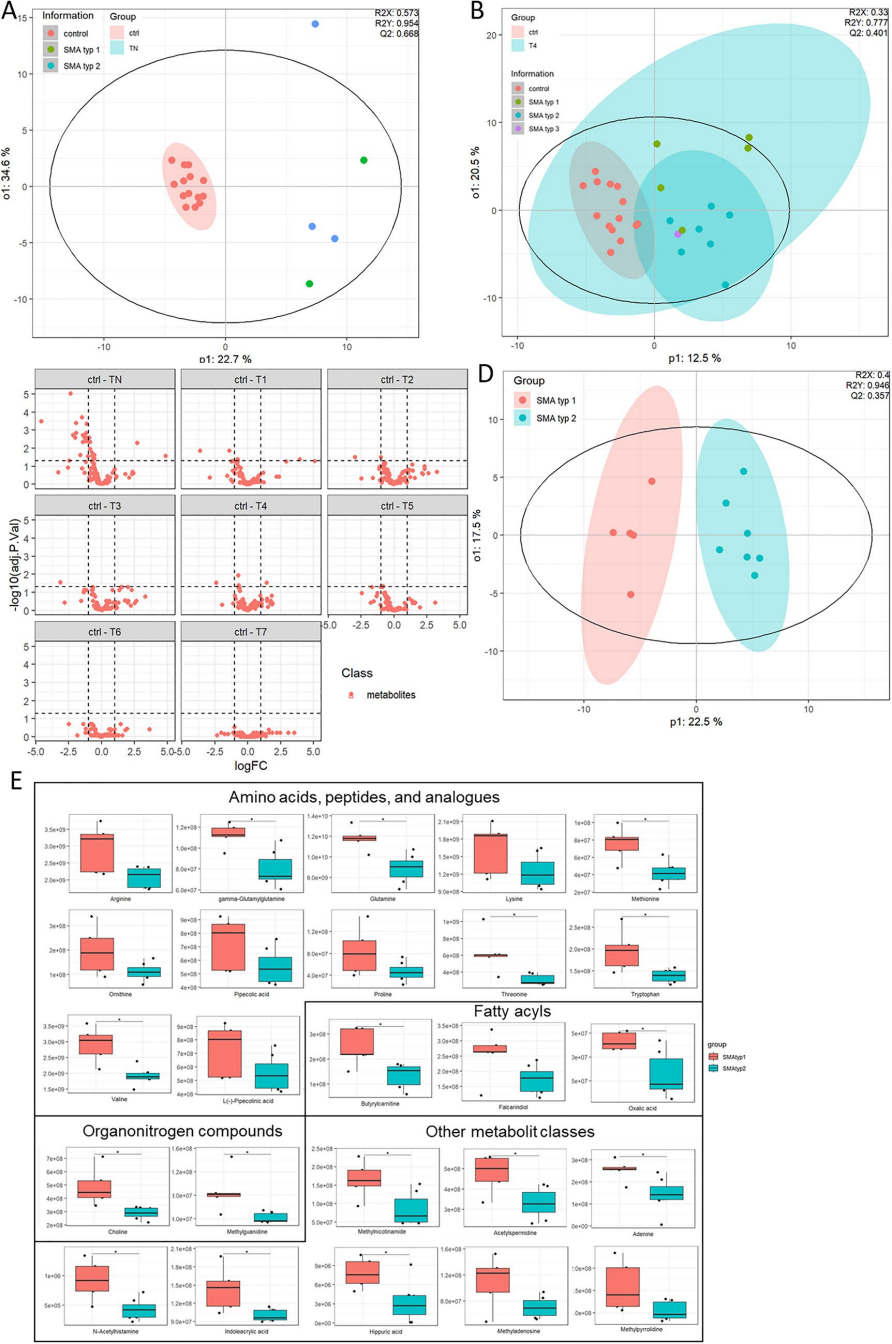
Multivariate analysis of metabolomics profiles obtained from CSF of study cohort. **A** OPLS-DA of therapy-naïve (TN) SMA patients compared to ctrl. **B** OPLS-DA of SMA patients under Nusinersen therapy (T4) compared to ctrl. **C** Volcano plot of metabolites identified in the CSF of SMA patients in the therapy-naïve state (TN) as well as under therapy (T1–T7). **D** OPLS-DA of SMA patients type I versus type II under Nusinersen therapy. **E** Univariate analysis comparing the CSF of type I and type II SMA patients focusing on the top 25 metabolites identified through OPLS-DA

**Fig. 6:**
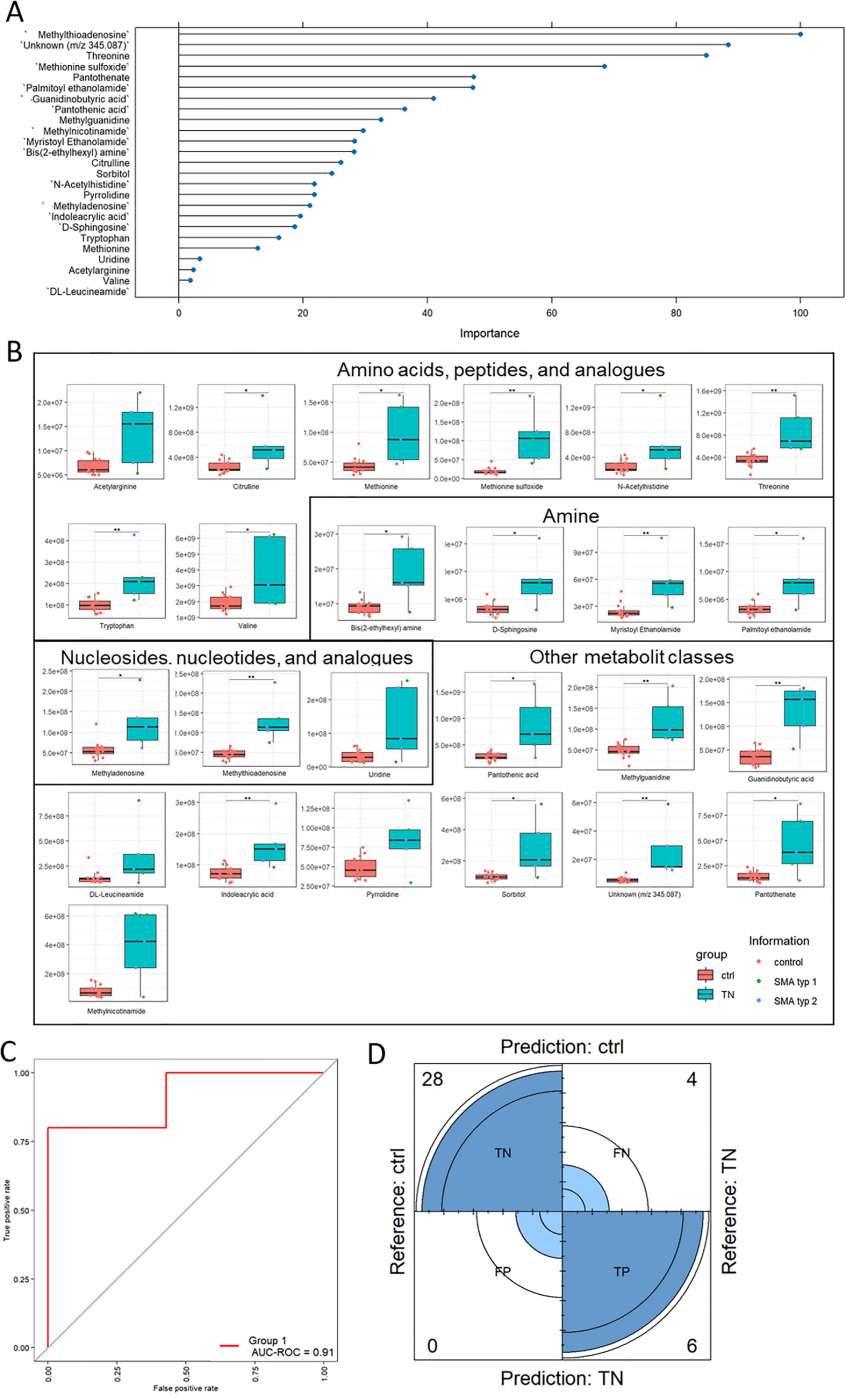
Univariate analysis of top 25 compounds identified through metabolomics analysis. **A** Ranking of the 25 most important metabolites responsible for altered CSF metabolome profile in TN SMA patients compared to ctrl. **B** These 25 metabolites were selected for univariate analysis using box plots. **C** ROC plot with an AUC of 0.91. **D** Confusion matrix: out of 38 cases, 28 cases were identified as true negative, six as true positive, four as false negative, and zero false positive

MVA revealed that in the CSF, all TN SMA patients including two type I and two type II patients were clearly separated from ctrl (Fig. 5A). Under Nusinersen therapy, this separation between SMA patients and the ctrl group was no longer detectable (Fig. 5B). This therapeutic effect could also be observed in the volcano plots of all metabolites identified. It was shown that, when comparing CSF from ctrl and TN patients, various metabolites were significantly up- and downregulated by twofold. Under therapy with Nusinersen, fewer metabolites were significantly altered with time point T7 having no siginificant alterations. An OPLS-DA between T4 SMA type I and type II patients revealed separation (Q3 value of 0.357). Among the top 25 hits, 12 metabolites were listed according to the HMDB in the category “amino acids, peptides and analogues”, two organonitrogen compounds, three fatty acyls, and eight metabolites belonging to other categories. Of note, in the UVA choline and the essential amino acids glutamine, methionine, threonine, tryptophan, and valine were significantly reduced in the SMA type II group (Fig. 6).

**Fig. 7:**
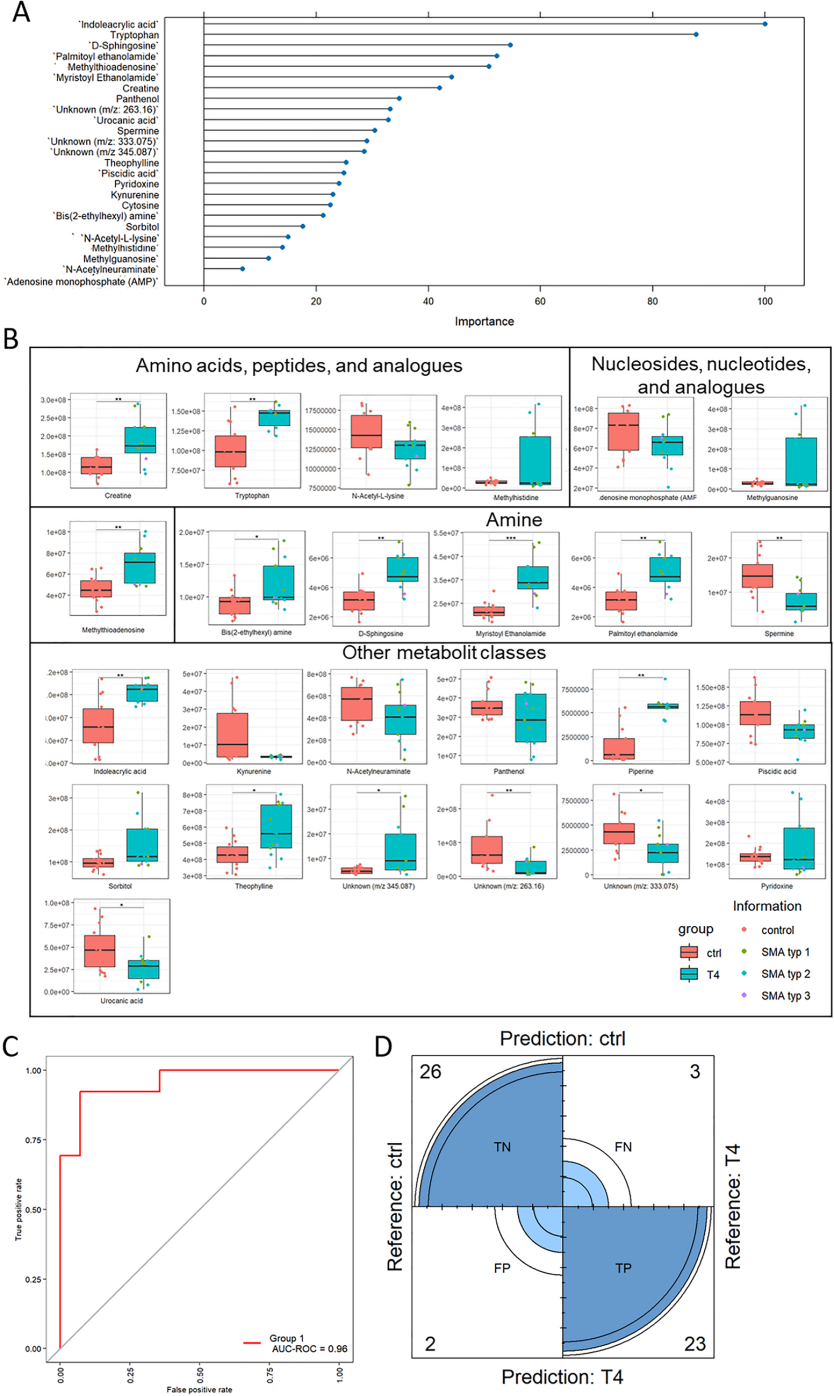
Univariate analysis of top 25 compounds identified through metabolomics analysis in the CSF of T4 SMA patients vs. ctrl. **A** VIP plot of top 25 metabolites according to molrank. **B** Univariate analysis using box plot. Red bars indicate ctrl and Turquoise bars indicate SMA patients including SMA type I (green dots), SMA type II (blue dots), and SMA type III (purple dot). **C** ROC plot with a calculated AUC-ROC of 0.96. **D** Confusion matrix generated predicted 26 cases as true negative (TN), 23 cases as true positive (TP), two false positives (FP), and three false negatives (FN)

To gain deeper insights into the metabolites present in the CSF of SMA patients compared to ctrl subjects, we conducted UVA of detected metabolites. In the CSF of TN SMA patients compared to ctrl our top 25 list included a diverse range of compounds. Interestingly, the amino acids citrulline, methionine, methionine sulfoxide, threonine, tryptophan, and valine were signficantly increased in the TN SMA group compared to ctrl. Amines related to the lipid metabolism, such as sphingosine, myristoylethanolamide, and palmitoylethanolamide, were also elevated in this patient group by at least ~ 1.9-fold compared to ctrl. The top hit was methylthioadenosine, a metabolite listed in the category “Nucleosides, nucleotides and analogues”. Both, methylthioadenosine and methyladenosine, also belonging to this category, were significantly increased by ~ 1.9-fold in the CSF of TN SMA patients compared to ctrl. To assess the performance of our model, we generated an ROC plot, which yielded an AUC-ROC value of 0.91. Additionally, our confusion matrix correctly predicted 28 cases as true negative and six cases as true positive, with no false positives and four false negatives (Fig. 7).

In the CSF of T4 SMA patients compared to ctrl subjects, we identified the top 25 compounds of OPLS-DA with indoleacrylic acid as the top hit being ~ twofold increased in the T4 SMA patients compared to ctrl. Among these compounds, we observed significantly increased levels of metabolites related to lipid metabolism, such as sphingosine, myristoylethanolamide, and palmitoylethanolamide by ~ twofold. In addition, compared to ctrl the creatine as well as tryptophan were significantly increased in the T4 SMA group ~ twofold and significantly decreased levels of the unknown features ‘m/z 345.087’ (by 2.0-fold). Although the separation observed in the OPLS-DA plot was not as distinct as that seen in TN patients compared to ctrl, the AUC-ROC value was at 0.96. The confusion matrix yielded the following predictions: 26 samples as true negative, 23 samples as true postive, 2 false positives, and 3 false negatives indicating good predictive power.

### Proteomics

**Fig. 8:**
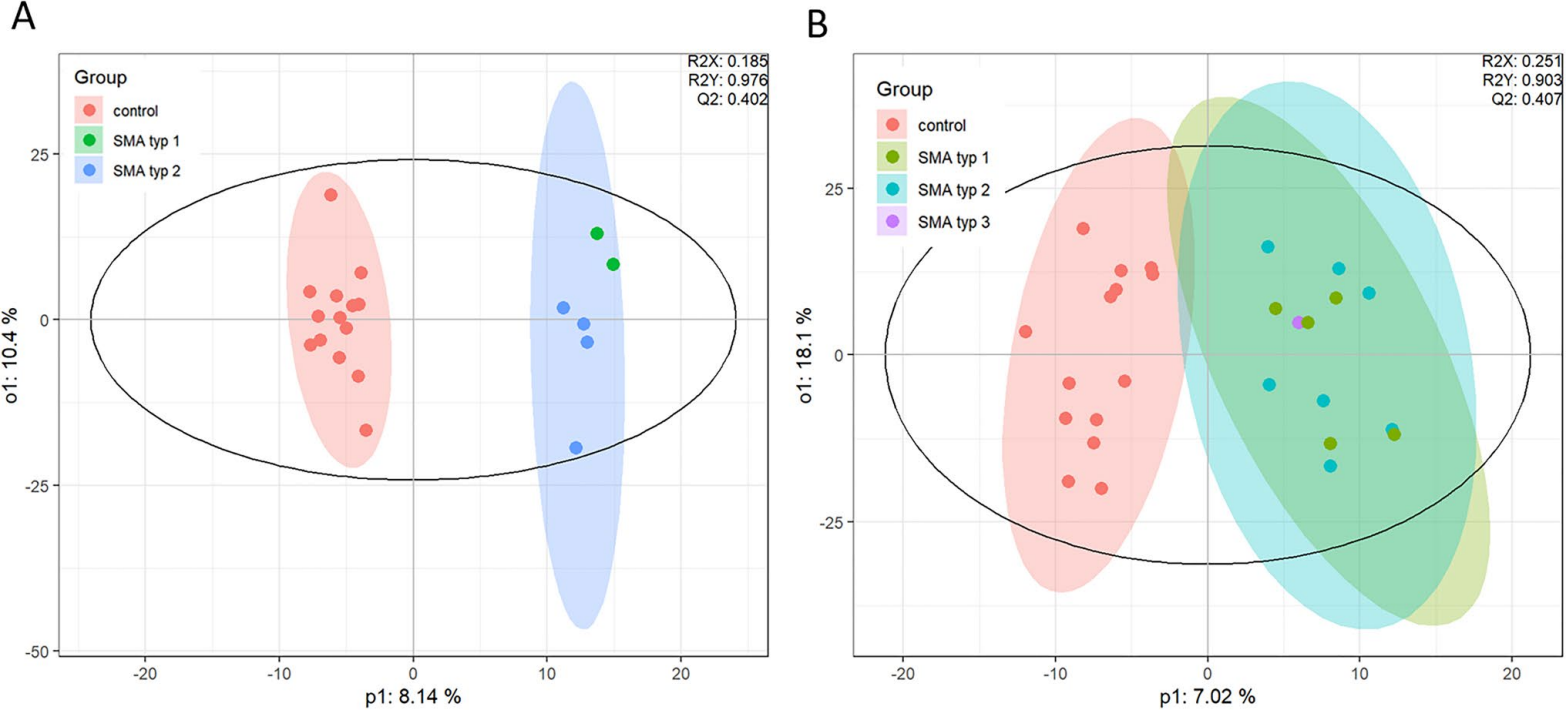
Proteomics analysis of CSF obtained from SMA patients and ctrl. **A, B** OPLS-DA of proteomics profiles obtained from the CSF of SMA patients in the therapy-naïve state (TN, **A**) as well as in response to Nusinersen treatment (T4, **B**)

Using our in-house proteomics method, we identified 997 proteins in the CSF of our study cohort. Similar to observations from lipidomics and metabolomics analyses, OPLS-DA demonstrated better separation in the CSF of TN SMA patients compared to ctrl than in T4 SMA patients compared to ctrl, indicating an approximation to a physiological protein profile through Nusinersen treament (Fig. 8A, B). However, volcano plots established for SMA patients in the TN state as well as in response to Nusinersen treatment compared to ctrl as well as for the CSF of T4 SMA patients compared to TN only showed trends but no statistically significant alterations of at least twofold (Supplement Fig. 1). Supplement Table 3 and 4 show the top 50 proteins identified in the CSF of TN SMA patients and T4 SMA patients versus ctrl, respectively.

## Discussion

In this extensive biomarker study, we demonstrate that the CSF multi-omics profiles of SMA patients differ significantly depending on whether patients are in the therapy-naïve state or undergoing therapy with the intrathecal anti-sense oligonucleotide Nusinersen. This effect was evident in the lipidomics and metabolomics profile, particularly in the generated volcano plots of metabolomics data, of these patients compared to sex- and age-matched ctrl. In TN patients compared to ctrl, many compounds were significantly altered. However, during a minimum of 6 months of therapy (equals timepoint T4 in this study), these changes diminished significantly. Although other studies have shown that later time points in therapy exhibit better therapeutic effects on the molecular level [14], we selected time point 4 for our “under therapy with Nusinersen” group, because the therapeutic effect was already observable by a change of several biomarkers and we had the largest sample size at this time point, ensuring statistically robust results.

To the best of our knowlegde, we demonstrate for the first time, through lipidomics analysis, a shift from CE to Chol, as well as from lyso-PL to PL in CSF from SMA patients compared to ctrl. This could indicate an altered HDL-like particle metabolism in the CNS of SMA patients. Since cholesterol metabolism in the CNS is separate from that of the periphery [27], the only lipoproteins present in the CNS are HDL-like particles. This shift was particularly evident in therapy naïve patients, but also to some extent in patients undergoing Nusinersen therapy, suggesting that while Nusinersen treatment is beneficial, it does not completely reverse metabolic lipid alterations. It has already been shown in the past that SMA is associated with dyslipidemia [16–18]. Similarly, Feng et al. demonstrated that SMA patients exhibit a different plasma lipid profile compared to ctrl in 56 lipids and lipid-like molecules [15]. When performing the CSF CEC assay, we observed that cholesterol efflux was drastically reduced in TN patients compared to ctrl. CEC was also significantly reduced in patients under therapy (T4), but notably increased compared to therapy naïve samples, indicating improved HDL function in response to Nusinersen. In line, MVA of proteomics data identified various proteins involved in lipid metabolism, particularly those important for lipoprotein metabolism as important discriminators (see Supplement Tables 3 and 5). We found increased levels of clusterin, LSR1, and LRP11 and decreased levels of apoF in the CSF of SMA patients under Nusinersen therapy compared to TN CSF, again indicating changed HDL function. Similiarly, when comparing TN CSF of SMA patients to ctrl, we identified reduced levels of LRP11 and prosaposin (PSAP), which are both involved in lipid metabolism by activating hydrolysis of sphingolipids [28]. Previously published literature has reported alterations in apolipoproteins of SMA patients under DMT, especially increased apoE and apoA1 levels [6]. In this study, we specify that CEC and thus HDL function is impaired in the CSF of SMA patients compared to ctrl, and that this impairment can be reversed by Nusinersen treatment. HDL-like particles in the CNS originate to a minor extent from peripheral blood, by crossing the blood–brain barrier via SRBI-mediated uptake and transcytosis [29, 30]. The major part of HDL-like particles in the brain are synthesized by glial cells and have central origin. These particles are crucial for maintaining lipid homeostasis in the CNS and play a major role in neuroprotection and neuronal health.

MVA of metabolomics data identified several important discriminators associated with lipid metabolism. Choline, for instance, the top dicriminating molecule when comparing CSF from SMA type I and II patients, was significantly reduced in type II patients compared to type I. Choline plays a central role in the CNS as a precursor for acetylcholine synthesis [31] and for the synthesis of PC and sphingomyelin [32]. In line, MVA of metabolomics data identified sphingosine to be decreased and as a top discriminator in both, the TN and T4 SMA groups compared to ctrl. Sphingosine serves in part as a precursor of sphingosine-1-phosphate, a lipid signaling molecule crucial for vascular development, and involved in various cellular processes, such as signal transduction, cell proliferation, apoptosis, and nervous system regulation [33, 34]. Furthermore, sphingosine plays a crucial role in sphingolipid synthesis, such as Cer and SM [35], which were found to be significantly reduced in the CSF of TN as well as T4 SMA patient group compared to ctrl. These findings indicate that sphingolipid metabolism remains impaired in SMA patients despite Nusinersen therapy, and retains patterns observed in other motor neuron diseases with no specific treatment available [36, 37].

Furthermore, our metabolomics analyses identified amino acids in the CSF as important discriminators between (1) SMA patients and ctrl, and (2) SMA types I and II. These findings align with previously published results, indicating that aromatic amino acids, such as histidine, tryptophan, tyrosine, and phenylalanine, are altered in the CSF of SMA patients [14, 38]. Faravelli et al. demonstrated that Nusinersen therapy in SMA type III led to increased levels of histidine, phenylalanine, tryptophan, and glutamine [14]. Valsecchi et al. identified methionine, tyrosine and ATP as discriminating molecules in the liver of SMN∆7 mice compared to wild-type mice [38]. Amino acids play a crucial role in the CNS and neuronal health, not only by being essential for protein synthesis, but also by functioning as neurotransmitters in neuronal connection and signal transmission [39]. Next to other neurotransmitter classes, such as amines, amino acids are among the most abundant neurotransmitters found in the CNS [40]. When comparing the CSF of T4 SMA type I and SMA type II patients, we identified 13 changed amino acids with glutamine levels increased only in the CSF of SMA type II patients, while other essential amino acids, such as arginine, lysine, methionine, ornithine, threonine, and valine, were downregulated. Glutamine serves as a precursor for glutamate and the neurotransmitter gamma-aminobutyric acid (GABA). GABA and glycine are the most abundant inhibitory neurotransmitters, leading to hyperpolarization of the postsynaptic membrane and producing inhibitory postsynaptic potentials (IPSP). Arginine is a precursor of the neurotransmitter nitric oxide, which is involved in vasodilation, synaptic plasticity, and neuroprotection.

When comparing the CSF of TN SMA patients with ctrl, we detected increases in eight “amino acids, peptides and analogues” according to HMDB, including significant increases in citrulline, methionine, methionine sulfoxide, *N*-acetylhistidine, threonine, tryptophan, and valine. Methionine not only shows antioxidant properties and functions as a precursor for the methyl donor *S*-adenosylmethionine (SAM), but it also plays a role in neurotransmitter synthesis, including dopamine and serotonin [41, 42]. In T4 SMA patients, compared to ctrl, only three amino acids were among the top discriminators, including significantly increased tryptophan. This confirms that Nusinersen is able to alter amino acid levels toward a physiological. Nevertheless, tryptophan levels were significantly increased in both SMA groups (TN and T4) compared to ctrl. Additionally, indoleacrylic acid, a metabolite derived from tryptophan associated with neuroinflammation and modulating immune response in the CNS [43], was identified as top discriminating molecule in the T4 SMA group versus ctrl. This is important because (1) the aromatic amino acid tryptophan is an important precursor for serotonin synthesis, affecting mood, (2) patients with hypertryptophanemia show neurological deficits, behavioral changes, impaired memory, and reduced IQ[44], and (3) the cognitive performance of several SMA type I patients is below average [45]. Furthermore, tryptophan is an essential substrate for the kynurenine pathway, which is connected to the pipecolic acid pathway for lysine metabolism through shared enzymes. This interconnection has also been associated with a neuronal developmental role as well as neurologic conditions such as schizophrenia [46].

As already described above, proteomics analysis identified 927 proteins in the CSF, albeit with no statistical significant differences between SMA patients and controls, probably due to low sample size of our study cohort. Nevertheless, our in-house analysis confirmed findings of previous studies by detecting various collagens, e.g., COL5A1, COL1A1, COL1A2, and COL11A1, to be top discriminators of SMA [47] (see supplement tables 3 and 4). In line with Kessler et al., we found an upregulation of Parkinson’s disease protein 7 (PARK7) [48], a chaperone that protects neurons by a-synuclein aggregation among the top 30 altered proteins in the loading plot in response to treatment with Nusinersen compared to TN SMA patients as well as ctrl.

## Conclusion

In summary, we performed extensive multi-omics profiling of CSF from SMA patients under treatment with Nusinersen, compared to age- and sex-matched ctrl. For the first time, we demonstrate an altered ratio of PL and their lysoforms as well as free Chol and CE, likely indicating a changed function of HDL-like particles in the CSF. At the molecular level, some of these alterations are at least partially reversible by Nusinersen therapy. Based on this study, we hypothesize that during Nusinersen therapy, neurons and neuronal function recovers, reducing the concentrations of amino acids in line with improved cell anabolism. Recently published results of clinical trials, including the ENDEAR and ongoing DEVOTE study, suggest that higher doses of Nusinersen treatment are not only well tolerated but achieve steady-state conditions of Nusinersen in the CSF at an earlier time point, leading to dose-dependent decreases in phosphorylated heavy-chain filaments, a biomarker corellating with disease severity [49, 50]. Future omics studies will help to show whether an adapted treatment protocol with higher doses of Nusinerse can further enhance its role as a potent disease modulator by fully restoring metabolic perturbations to a physiologic state.

## Supplementary Information

Below is the link to the electronic supplementary material.

Supplementary material 1 (DOCX 98 KB)

## Data Availability

The data presented in this study as well as markdowns of the respective R scripts are openly available at Metabolomics Workbench (Project title: “Multiomics profiling in Spinal Muscular Atrophy SMA): Insights from longitudinal CSF analysis of patients under treatment with Nusinersen”). Raw data are provided as “.raw” files, and data processed with LDA are available in excel files. An additional excel file named “metadata” provides sample information.
