# Supplementary material for: Multi-omics profiling in spinal muscular atrophy (SMA): investigating lipid and metabolic alterations through longitudinal CSF analysis of Nusinersen-treated patients
Source: J Neurol. 2025 Feb 4;272(3):183. doi: 10.1007/s00415-025-12909-4 (PMC11794407; doi:10.1007/s00415-025-12909-4)
Supplement: Supplementary file 1 — Supplementary material 1 (DOCX 98 KB) [file 415_2025_12909_MOESM1_ESM.docx]

**Supplement**

**Supplement Table 1: SMN copy number of patient cohort included in the study**

|  | Patient/Gender | Age at TN/T4 [months] | SMA type | SMN Copy Number |
| --- | --- | --- | --- | --- |
| 1 | TN/f | 1 | I | 3 |
| 2 | T4/m | 12 | I | 3 |
| 3 | TN/f | 22 | II | 3 |
| 4 | TN/m | 36 | II | 3 |
| 5 | TN/f | 11 | II | 3 |
| 6 | TN/m | 5 | I | 2 |
| 7 | T4/f | 62 | I | 2 |
| 8 | TN/f | 168 | II | 4 |
| 9 | T4/m | 185 | II | 3 |
| 10 | T4/m | 60 | III | 6 |
| 11 | T4/f | 75 | I | 2 |
| 12 | T4/f | 61 | II | 4 |
| 13 | T4/m | 115 | II | 3 |

**Supplement Table 2: List of metabolites identified through metabolomics analysis and classification into confidence levels**

|  | **RT[min]** | **m/z** | **Compound** | **Confidence Level** |
| --- | --- | --- | --- | --- |
| 1 | 2.90 | 61.0396 | **Urea** | 1 |
| 2 | 10.70 | 72.081 | **Pyrrolidine** | 2 |
| 3 | 11.10 | 74.061 | **Methylguanidine** | 2 |
| 4 | 11.30 | 76.0393 | **Glycine** | 1 |
| 5 | 6.60 | 76.076 | **Trimethylamine N-oxide** | 2 |
| 6 | 1.50 | 100.076 | **Methylpyrrolidine** | 2 |
| 7 | 10.90 | 90.055 | **Alanine** | 1 |
| 8 | 9.90 | 91.003 | **Oxalic acid** | 2 |
| 9 | 9.50 | 86.097 | **N-Methylpyrrolidone** | 2 |
| 10 | 2.90 | 102.055 | **Aminocyclopropanecarboxylate** | 2 |
| 11 | 2.00 | 104.071 | **Aminoisobutyric acid** | 2 |
| 12 | 6.70 | 104.107 | **Choline** | 1 |
| 13 | 11.70 | 106.0499 | **Serine** | 1 |
| 14 | 11.70 | 108.0566 | **Serine 2,3 C13** | 1 |
| 15 | 7.60 | 112.051 | **Cytosine** | 2 |
| 16 | 11.60 | 112.0869 | **Histamine** | 1 |
| 17 | 5.50 | 114.0662 | **Creatinine** | 1 |
| 18 | 13.20 | 116.0706 | **Proline** | 1 |
| 19 | 9.80 | 118.0863 | **Valine** | 1 |
| 20 | 11.20 | 120.0655 | **Threonine** | 1 |
| 21 | 3.60 | 139.05 | **Urocanic acid** | 2 |
| 22 | 14.30 | 122.027 | **Cysteine** | 1 |
| 23 | 7.60 | 123.0553 | **Nicotinamide** | 1 |
| 24 | 10.50 | 127.05 | **Imidazoleacetic acid** | 2 |
| 25 | 11.60 | 130.05 | **L-Pyroglutamic acid** | 1 |
| 26 | 13.00 | 130.086 | **Pipecolic acid** | 2 |
| 27 | 13.10 | 130.086 | **L(-)-Pipecolinic acid** | 2 |
| 28 | 9.40 | 131.118 | **DL-Leucineamide** | 2 |
| 29 | 9.70 | 132.1019 | **Isoleucine** | 1 |
| 30 | 9.40 | 132.1019 | **Leucine** | 1 |
| 31 | 11.70 | 133.0608 | **Asparagine** | 1 |
| 32 | 11.80 | 133.061 | **Ureidopropionic acid** | 2 |
| 33 | 13.20 | 133.0972 | **Ornithine** | 1 |
| 34 | 12.20 | 134.0448 | **Aspartate** | 1 |
| 35 | 5.10 | 136.0617 | **Adenine** | 1 |
| 36 | 5.90 | 137.046 | **Hypoxanthine** | 2 |
| 37 | 8.10 | 137.071 | **Methylnicotinamide** | 2 |
| 38 | 10.50 | 141.066 | **Methylimidazoleacetic acid** | 2 |
| 39 | 9.60 | 144.102 | **Proline betaine** | 2 |
| 40 | 2.30 | 146.081 | **Acetamidobutyric acid** | 2 |
| 41 | 2.30 | 146.092 | **Guanidinobutyric acid** | 2 |
| 42 | 8.90 | 146.1181 | **Acetylcholine** | 1 |
| 43 | 14.40 | 146.1652 | **Spermidine** | 1 |
| 44 | 11.60 | 147.0764 | **Glutamine** | 1 |
| 45 | 13.10 | 147.1128 | **Lysine** | 1 |
| 46 | 11.20 | 148.0604 | **Glutamate** | 1 |
| 47 | 3.10 | 150.0583 | **Methionine** | 1 |
| 48 | 2.30 | 154.05 | **Hydroxyanthranilic acid** | 2 |
| 49 | 10.80 | 132.0768 | **Creatine** | 1 |
| 50 | 1.50 | 154.086 | **Dopamine** | 2 |
| 51 | 8.20 | 154.097 | **N-Acetylhistamine** | 2 |
| 52 | 12.90 | 156.0768 | **Histidine** | 1 |
| 53 | 11.80 | 162.076 | **L-Aminoadipic acid** | 2 |
| 54 | 10.40 | 162.1125 | **Carnitine** | 1 |
| 55 | 11.60 | 166.0532 | **Methionine sulfoxide** | 1 |
| 56 | 9.30 | 166.0863 | **Phenylalanine** | 1 |
| 57 | 2.10 | 168.065 | **Pyridoxal** | 2 |
| 58 | 11.70 | 169.058 | **Orsellinic acid** | 2 |
| 59 | 3.70 | 170.082 | **Pyridoxine** | 2 |
| 60 | 12.20 | 170.092 | **3-Methylhistidine** | 2 |
| 61 | 12.90 | 175.119 | **Arginine** | 1 |
| 62 | 11.90 | 176.103 | **Citrulline** | 1 |
| 63 | 11.20 | 180.66 | **Hippuric acid** | 2 |
| 64 | 1.60 | 181.070 | **Glucose** | 1 |
| 65 | 10.30 | 182.0812 | **Tyrosine** | 1 |
| 66 | 2.30 | 182.19 | **Dicyclohexylamine** | 2 |
| 67 | 9.20 | 188.074 | **Indoleacrylic acid** | 2 |
| 68 | 12.20 | 188.176 | **Acetylspermidine** | 2 |
| 69 | 9.10 | 189.088 | **N-Acetyl-L-glutamine** | 2 |
| 70 | 12.60 | 189.123 | **N-Acetyl-L-lysine** | 2 |
| 71 | 5.30 | 192.159 | **Triisopropanolamine** | 2 |
| 72 | 11.00 | 198.087 | **N-Acetylhistidine** | 2 |
| 73 | 6.90 | 202.18 | **Unknown (m/z 202.18)** | 3 |
| 74 | 10.50 | 203.052 | **Theophylline** | 2 |
| 75 | 12.10 | 203.15 | **N,N-Dimethylarginine** | 2 |
| 76 | 9.40 | 203.223 | **Spermine** | 2 |
| 77 | 8.80 | 204.113 | **Aminoantipyrine** | 2 |
| 78 | 8.80 | 204.123 | **Acetylcarnitine** | 1 |
| 79 | 10.40 | 205.068 | **Sorbitol** | 1 |
| 80 | 9.20 | 205.0972 | **Tryptophan** | 1 |
| 81 | 1.90 | 206.138 | **Panthenol** | 2 |
| 82 | 9.20 | 209.0921 | **Kynurenine** | 2 |
| 83 | 10.80 | 217.129 | **Acetylarginine** | 2 |
| 84 | 12.10 | 217.155 | **Unknown (m/z: 217.155)** | 3 |
| 85 | 8.00 | 218.138 | **Propionylcarnitine** | 2 |
| 86 | 3.10 | 220.118 | **Pantothenic acid** | 2 |
| 87 | 5.50 | 227.1139 | **Carnosine** | 1 |
| 88 | 8.30 | 231.084 | **Benzanthrone** | 2 |
| 89 | 7.00 | 232.154 | **Butyrylcarnitine** | 2 |
| 90 | 10 | 241.154 | **Unknown (m/z: 241.154)** | 3 |
| 91 | 3.50 | 242.1 | **Pantothenate** | 2 |
| 92 | 1.60 | 247.107 | **N-Acetyl tryptophan** | 2 |
| 93 | 1.50 | 242.284 | **Bis(ethylhexyl) amine** | 2 |
| 94 | 9.90 | 244.079 | **N-Acetylgalactosamine** | 2 |
| 95 | 9.90 | 244.093 | **Cytidine** | 2 |
| 96 | 10.70 | 245.0768 | **Uridine** | 1 |
| 97 | 12.60 | 246.17 | **Valerylcarnitine** | 2 |
| 98 | 7.30 | 247.144 | **Tryptophan betaine** | 2 |
| 99 | 9.20 | 257.074 | **Piscidic acid** | 2 |
| 100 | 10.70 | 261.181 | **Falcarindiol** | 2 |
| 101 | 10.9 | 263.16 | **Unknown (m/z: 263.16)** | 3 |
| 102 | 7.30 | 265.1118 | **Thiamine** | 1 |
| 103 | 6.10 | 268.104 | **Adenosine** | 1 |
| 104 | 2.30 | 272.258 | **Myristoyl Ethanolamide** | 2 |
| 105 | 12.10 | 332.095 | **N-Acetylneuraminate** | 2 |
| 106 | 13.00 | 276.119 | **gamma-Glutamylglutamine** | 2 |
| 107 | 10.70 | 277.103 | **gamma-Glutamylglutamic acid** | 2 |
| 108 | 1.10 | 277.139 | **Saccharopine** | 2 |
| 109 | 9.90 | 282.119 | **Methyladenosine** | 2 |
| 110 | 11.70 | 287.237 | **Retinol** | 2 |
| 111 | 2.30 | 298.097 | **Methylthioadenosine** | 2 |
| 112 | 7.60 | 298.115 | **Methylguanosine** | 2 |
| 113 | 1.90 | 300.289 | **Palmitoyl ethanolamide** | 2 |
| 114 | 2.00 | 300.289 | **D-Sphingosine** | 2 |
| 115 | 10.60 | 305.098 | **N-Acetylaspartylglutamic acid** | 2 |
| 116 | 2.50 | 308.118 | **Piperine** | 2 |
| 117 | 12.10 | 310.113 | **Sialic acid** | 2 |
| 118 | 3.10 | 316.248 | **Decanoylcarnitine** | 1 |
| 119 | 7.6 | 333.075 | **Unknown (m/z: 333.075)** | 3 |
| 120 | 13.3 | 336.140 | **Unknown (m/z: 336.140)** | 3 |
| 121 | 9.3 | 345.087 | **Unknown (m/z 345.087)** | 3 |
| 122 | 12.10 | 348.070 | **Adenosine monophosphate (AMP)** | 1 |
| 123 | 9.5 | 265.118 | **Phenylacetylglutamine** | 2 |

**Supplement Table 3: Top 50 proteins (molrank) identified in the CSF of ctrl vs. TN SMA Patients**

|  | **Protein_ID** | **p1** | **o1** | **molrank** |
| --- | --- | --- | --- | --- |
| 1 | NTM | -0.00002968952030035699 | -0.07650452514843521 | 972 |
| 2 | LUM | 0.00009396780766892023 | -0.06218392498869236 | 971 |
| 3 | PCDH8 | -0.00009600434687848555 | -0.008813377757937479 | 970 |
| 4 | CTSB | 0.000155414953436022 | -0.006041268831313167 | 969 |
| 5 | VDAC2 | -0.0001555342414058897 | 0.008039843772599406 | 968 |
| 6 | CHAD | 0.0002063641665445847 | 0.04065407808969566 | 967 |
| 7 | HABP2 | 0.0002366443030493104 | 0.02105142009417627 | 966 |
| 8 | CRNN | -0.0003088352012837299 | 0.0102061676164605 | 965 |
| 9 | COL1A1 | 0.0003290224694322477 | -0.02770030241703639 | 964 |
| 10 | IGKV1-27 | 0.0003674530473438301 | -0.04267137444389572 | 963 |
| 11 | CNTN6 | 0.0003756681024710052 | 0.009900263208695222 | 962 |
| 12 | CTBS | -0.0003959057797233757 | 0.02677944491088764 | 961 |
| 13 | NUCB1 | -0.0004000981036327717 | -0.01592414589420209 | 960 |
| 14 | PCP4 | -0.0004705752785780767 | 0.08891601852626052 | 959 |
| 15 | SDCBP | -0.0005221136935924748 | 0.01311036510673124 | 958 |
| 16 | SIGLEC14 | -0.0005336756550770773 | -0.05726989265121021 | 957 |
| 17 | LRP11 | -0.000623722486995089 | -0.0106267251236573 | 956 |
| 18 | HEG1 | 0.0006382241527558792 | 0.05289083154007709 | 955 |
| 19 | LTBP1 | 0.0007951697860184195 | -0.03017059332191426 | 954 |
| 20 | NRP2 | -0.000817953768724517 | 0.04241631434172785 | 953 |
| 21 | MLEC | 0.0008323660634662853 | -0.0360809354331625 | 952 |
| 22 | KLK6 | 0.0009058505756118975 | 0.05355186494094663 | 951 |
| 23 | RTBDN | -0.0009837336841095592 | -0.03243586530374797 | 950 |
| 24 | CDH10 | -0.0009850139572891725 | -0.02791419984570578 | 949 |
| 25 | RPL5 | 0.001066806644564823 | 0.05395326569869727 | 948 |
| 26 | CTSA | 0.001094906663237854 | -0.03433281049531548 | 947 |
| 27 | IL1RN | -0.001144088767561248 | -0.05341145081073711 | 946 |
| 28 | SLC1A2 | -0.001173505856407484 | 0.01583112118256051 | 945 |
| 29 | KHDRBS1 | -0.001437234245205828 | -0.06672137068952702 | 944 |
| 30 | GDI1 | 0.001564566566230105 | 0.01427311093679636 | 943 |
| 31 | ATP12A | -0.001721437311324452 | -0.04741276122294966 | 942 |
| 32 | CP | 0.001901858897471784 | 0.05718524310785865 | 941 |
| 33 | IGKV3D-20 | -0.0019315691843286 | 0.01408644272402805 | 940 |
| 34 | CANT1 | -0.00195610194756351 | -0.04160827854168624 | 939 |
| 35 | PSAP | -0.002039821453022728 | -0.008865650871363663 | 938 |
| 36 | CPB2 | 0.002059856638123763 | -0.008574524123752215 | 937 |
| 37 | SHISA5 | -0.002071666069353298 | -0.03479507564190055 | 936 |
| 38 | MEGF8 | 0.002130937011998679 | -0.04796346140108435 | 935 |
| 39 | MAN2A1 | 0.002166210227288736 | 0.03995009730533826 | 934 |
| 40 | MIA | -0.002213337690970608 | -0.05849860370433222 | 933 |
| 41 | CANX | 0.00221361582021208 | 0.006541485422183372 | 932 |
| 42 | GAP43 | -0.002216173455513806 | -0.05777229869233223 | 931 |
| 43 | PTPRD | -0.002270600178580409 | 0.001112632443949157 | 930 |
| 44 | CRABP1 | 0.002278984860298083 | -0.03744524097253382 | 929 |
| 45 | FUCA1 | 0.002372584010281319 | -0.00591869736409688 | 928 |
| 46 | CNTN4 | 0.002383163815914064 | 0.0709274915653623 | 927 |
| 47 | MARCKS | 0.00240592216702789 | 0.06399096787026053 | 926 |
| 48 | PGAM1 | 0.002516541933403408 | -0.0092525857655969 | 925 |
| 49 | MANBA | 0.002599850105864123 | 0.006511243939163113 | 924 |
| 50 | MDH2 | -0.002620943074210996 | -0.01445990375143131 | 923 |

**Supplement Table 4: Top 50 proteins (molrank) identified in the CSF of ctrl vs. T4 SMA Patients**

|  | **Protein_ID** | **p1** | **o1** | **molrank** |
| --- | --- | --- | --- | --- |
| 1 | C1QA | 0,000062752 | 0,077754490 | 972 |
| 2 | SERPINB12 | -0,000086103 | 0,020230550 | 971 |
| 3 | CSTB | 0,000138075 | -0,073136839 | 970 |
| 4 | PCDH7 | 0,000187576 | -0,010998204 | 969 |
| 5 | SFN | 0,000268328 | -0,002533746 | 968 |
| 6 | KLK6 | -0,000273578 | 0,026455939 | 967 |
| 7 | LMNA | -0,000351487 | 0,002794389 | 966 |
| 8 | COL1A2 | -0,000360079 | 0,063846048 | 965 |
| 9 | ART3 | 0,000438000 | -0,004093298 | 964 |
| 10 | EPB41 | -0,000473120 | -0,001132403 | 963 |
| 11 | CDH4 | 0,000474146 | -0,027015246 | 962 |
| 12 | SCG2 | 0,000518905 | -0,003049475 | 961 |
| 13 | SELENBP1 | -0,000519577 | -0,021930477 | 960 |
| 14 | CTSA | 0,000539342 | -0,029052180 | 959 |
| 15 | NPDC1 | -0,000552088 | 0,016963512 | 958 |
| 16 | RAN | -0,000625697 | 0,028763126 | 957 |
| 17 | CHAD | -0,000650543 | 0,006916578 | 956 |
| 18 | OSCAR | -0,000678264 | 0,021523405 | 955 |
| 19 | PLXDC1 | -0,000688865 | 0,074409514 | 954 |
| 20 | CPQ | -0,000706973 | -0,018816868 | 953 |
| 21 | PTPRR | -0,000790395 | 0,035104037 | 952 |
| 22 | CDH10 | 0,000865841 | -0,000992362 | 951 |
| 23 | HSP90B1 | -0,000884287 | 0,019015799 | 950 |
| 24 | LDHB | 0,000939399 | 0,026115468 | 949 |
| 25 | NUCB1 | -0,001011466 | 0,039688060 | 948 |
| 26 | DCC | -0,001019020 | -0,062440393 | 947 |
| 27 | FSTL5 | -0,001055260 | -0,065063486 | 946 |
| 28 | PCDHAC2 | 0,001147327 | 0,021561191 | 945 |
| 29 | PCSK2 | 0,001151398 | -0,068310542 | 944 |
| 30 | CDH2 | 0,001270924 | 0,069947927 | 943 |
| 31 | YWHAE | 0,001275675 | 0,042451639 | 942 |
| 32 | PRKCSH | 0,001294434 | 0,037299775 | 941 |
| 33 | FKBP1A | -0,001365081 | -0,036314407 | 940 |
| 34 | SKP1 | 0,001534593 | 0,016579739 | 939 |
| 35 | IGFALS | 0,001566861 | -0,069278571 | 938 |
| 36 | IGHG3 | -0,001658534 | -0,028781784 | 937 |
| 37 | IGFBP7 | -0,001661704 | 0,037485065 | 936 |
| 38 | DSC1 | 0,001737662 | 0,014901096 | 935 |
| 39 | COL11A1 | 0,001776280 | 0,016892902 | 934 |
| 40 | LYVE1 | -0,001804721 | -0,005370331 | 933 |
| 41 | SDCBP | -0,001843949 | -0,010265249 | 932 |
| 42 | CASP14 | 0,001857910 | -0,024800154 | 931 |
| 43 | TGFBI | -0,001868259 | 0,009871919 | 930 |
| 44 | CSF1 | 0,001910990 | -0,011205318 | 929 |
| 45 | LTBP1 | -0,001915442 | 0,007701378 | 928 |
| 46 | CALY | -0,001944929 | -0,011121446 | 927 |
| 47 | PNP | 0,001993140 | -0,011908880 | 926 |
| 48 | ARPC3 | -0,002003030 | 0,060802436 | 925 |
| 49 | ITIH5 | -0,002058240 | 0,054510677 | 924 |
| 50 | POMGNT1 | -0,002082308 | 0,022498617 | 923 |

**Supplement Table 5: Top 50 proteins (molrank) identified in the CSF of TN SMA Patients vs. T4 SMA Patients**

|  | **Protein_ID** | **p1** | **o1** | **molrank** |
| --- | --- | --- | --- | --- |
| 1 | MAN2A1 | -4,62128E-05 | -0,03859 | 972 |
| 2 | ORM2 | 4,76543E-05 | -0,0168663 | 971 |
| 3 | CLU | 0,000126144 | 0,01561671 | 970 |
| 4 | IL6ST | 0,000150144 | -0,0049733 | 969 |
| 5 | LY6H | 0,000194426 | -0,0133688 | 968 |
| 6 | PDYN | -0,000275106 | 0,00202437 | 967 |
| 7 | PRKCSH | -0,000288914 | -0,0349102 | 966 |
| 8 | NAPRT | -0,000295225 | -0,0557019 | 965 |
| 9 | PIP | -0,000355218 | 0,01186157 | 964 |
| 10 | ART3 | -0,000372468 | -0,0028256 | 963 |
| 11 | CBLN4 | -0,000473933 | 0,03535344 | 962 |
| 12 | ACTBL2 | 0,000543113 | 0,05694918 | 961 |
| 13 | HBA1 | -0,000554131 | -0,0197903 | 960 |
| 14 | CDH13 | -0,000644521 | 0,04349544 | 959 |
| 15 | AHCY | -0,00072676 | 0,04382062 | 958 |
| 16 | BCAN | -0,000735159 | -0,0140051 | 957 |
| 17 | SERPINI1 | 0,000744883 | -0,0398605 | 956 |
| 18 | NLGN1 | -0,000765664 | -0,0434908 | 955 |
| 19 | LSR | 0,000841576 | 0,06114319 | 954 |
| 20 | ANK1 | -0,000887395 | -0,0253014 | 953 |
| 21 | GDI2 | -0,000965825 | 0,02032887 | 952 |
| 22 | ACTG1 | -0,000976015 | 0,02620972 | 951 |
| 23 | AXL | -0,001008838 | -0,0660951 | 950 |
| 24 | HSPA6 | -0,001022119 | -0,0052207 | 949 |
| 25 | APOF | -0,001083714 | -0,0192079 | 948 |
| 26 | CTBS | 0,001118028 | 0,01276087 | 947 |
| 27 | NRN1 | 0,001129972 | -0,0453784 | 946 |
| 28 | ITIH4 | -0,001149115 | -0,0415939 | 945 |
| 29 | PARK7 | 0,001151066 | -0,0316445 | 944 |
| 30 | ISLR2 | 0,001167122 | -0,0493016 | 943 |
| 31 | PSMA3 | -0,001181468 | -0,0206797 | 942 |
| 32 | PEBP1 | -0,001252671 | -0,0272933 | 941 |
| 33 | NUCB1 | -0,001318324 | -0,0321571 | 940 |
| 34 | HSPA5 | 0,001456118 | 0,02586672 | 939 |
| 35 | TGM1 | 0,001473502 | 0,00665366 | 938 |
| 36 | EIF4A1 | 0,001688926 | 0,0201364 | 937 |
| 37 | SERPINF2 | -0,001724842 | -0,0225396 | 936 |
| 38 | LRP11 | 0,00172833 | -0,0438578 | 935 |
| 39 | IGHG1 | -0,001743343 | -0,0617593 | 934 |
| 40 | CDH15 | -0,001819355 | 0,04261 | 933 |
| 41 | THY1 | -0,001910336 | -0,0446332 | 932 |
| 42 | A2M | -0,001923905 | 0,03662775 | 931 |
| 43 | C1QTNF4 | -0,001938768 | -0,0236641 | 930 |
| 44 | CECR1 | -0,002001002 | 0,01531452 | 929 |
| 45 | NUP85 | -0,002093244 | -0,0128459 | 928 |
| 46 | CP | 0,00218896 | -0,0085713 | 927 |
| 47 | MEGF9 | 0,002228455 | -0,0132321 | 926 |
| 48 | ARF1 | 0,002251026 | -0,0532049 | 925 |
| 49 | UBE2V2 | 0,002263394 | -0,0080431 | 924 |
| 50 | CHGB | 0,002273684 | 0,02196575 | 923 |


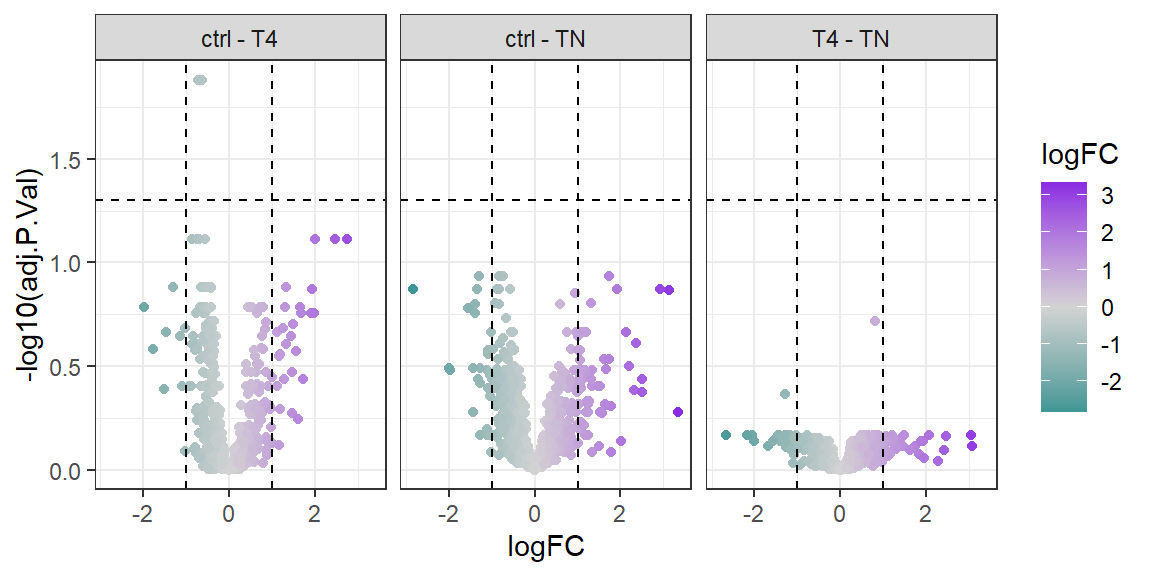


**Supplement Figure 1: Volcano plot of proteomics data obtained from CSF of SMA patients (TN and T4) and ctrl.**
